# *Stieleria tagensis* sp. nov., a novel member of the phylum *Planctomycetota* isolated from Tagus River in Portugal

**DOI:** 10.1007/s10482-023-01877-2

**Published:** 2023-09-22

**Authors:** Ofélia Godinho, Dominika Klimek, Adrianna Jackiewicz, Bárbara Guedes, Eduarda Almeida, Rita Calisto, Inês Rosado Vitorino, José Diogo Neves Santos, Ignacio González, Alexandre Lobo-da-Cunha, Magdalena Calusinska, Sandra Quinteira, Olga Maria Lage

**Affiliations:** 1 Departamento de Biologia, Faculdade de Ciências, Universidade do Porto, Rua do Campo Alegre s/n, 4169-007 Porto, Portugal; 2 CIMAR/CIIMAR, Centro Interdisciplinar de Investigação Marinha e Ambiental, Universidade do Porto, Terminal de Cruzeiros de Leixões, Av. General Norton de Matos s/n, 4450-208 Matosinhos, Portugal; 3 Environmental Research and Innovation Department, Luxembourg Institute of Science and Technology, Rue du Brill 41, 4422 Belvaux, Luxembourg; 4 The Faculty of Science, Technology and Medicine, FSTM, University of Luxembourg, 2 Avenue de l’Université, 4365 Esch-sur-Alzette, Luxembourg; 5 Centro de Excelencia en Investigación de Medicamentos Innovadores en Andalucía, Fundación MEDINA, 18016 Granada, Spain; 6 Laboratório de Biologia Celular, Instituto de Ciências Biomédicas Abel Salazar, ICBAS, Universidade do Porto, Rua de Jorge Viterbo Ferreira, 228, 4050-313 Porto, Portugal; 7 BIOPOLIS/CIBIO-InBIO, Centro de Investigação em Biodiversidade e Recursos Genéticos, Universidade do Porto, Rua Padre Armando Quintas, nº 7, 4485-661 Vairão, Portugal; 8 TOXRUN-Toxicology Research Unit, University Institute of Health Sciences, CESPU, CRL., Avenida Central de Gandra, 1317, 4585-116 Gandra, PRD Portugal

**Keywords:** *Planctomycetota*, Mesophilic aerobic bacterium, Genomic identification, *Pirellulaceae*, Brackish water

## Abstract

A bacterial strain was isolated from a brackish water sample of Tagus river, Alcochete, Portugal and was designated TO1_6^T^. It forms light pink colonies on M13 medium supplemented with *N*-acetylglucosamine. Cells are pear-shaped to spherical, form rosettes and divide by budding. Strain TO1_6^T^ presents a mesophilic and neutrophilic profile, with optimum growth at 20 to 25 °C and pH 7.0 to 7.5, and vitamin supplementation is not required to promote its growth. The genome of the novel isolate is 7.77 Mbp in size and has a DNA G + C content of 56.3%. Based on its 16S rRNA gene sequence, this strain is affiliated with the phylum *Planctomycetota*. Further taxonomic characterization using additional phylogenetic markers, namely *rpoB* gene sequence (encoding the β-subunit of the DNA-dependent RNA polymerase), as well as Percentage of conserved proteins, average nucleotide identity and average amino acid identity, suggest the affiliation of strain TO1_6^T^ to the genus *Stieleria*, a recently described taxon in the family *Pirellulaceae,* order *Pirellulales* and class *Planctomycetia*. Based on the genotypic, phylogenetic and physiological characterization, we here describe a new species represented by the type strain TO1_6^T^ (= CECT 30432^T^, = LMG 32465^T^), for which the name *Stieleria tagensis* sp. nov. is proposed.

**Supplementary Information:**

The online version contains supplementary material available at 10.1007/s10482-023-01877-2.

## Introduction

*Planctomycetota* is a phylum of Gram-negative bacteria that have received particular attention over the last decades, *e.g*. due to an increasing evidence of their bioactive potential, *i.e.* the production of antimicrobial and anticancer compounds (Calisto et al. 2019; Gimranov et al. 2022; Graca et al. 2016; Jeske et al. 2013, 2016; Sandargo et al. 2020) and of their potential as supplementary food source for microcrustaceans (Marinho et al. 2018, 2019). Additionally, their peculiar characteristics such as macromolecule uptake (Boedeker et al. 2017; Lonhienne et al. 2010), complex cellular ultrastructure (Boedeker et al. 2017; Santarella-Mellwig et al. 2010, 2013), broad-range antibiotic resistance (Cayrou et al. 2010; Godinho et al. 2019; Ivanova et al. 2021) and the unusual *ftsZ*-independent cell division (Jogler et al. 2012; Rivas-Marin et al. 2016; Rivas-Marin et al. 2020) also reinforces the interest in the study of fundamental biology within this phylum. Members of this phylum have been described across many environments, from freshwater (Kohn et al. 2020) to deep-sea hydrothermal deposits (Storesund et al. 2018; Storesund and Ovreas 2013) or boreal and subarctic wetlands (Dedysh and Ivanova 2019), associated with macroalgae (Bondoso et al. 2014, 2017; Lage and Bondoso 2011, 2014) and sponges (Izumi et al. 2013; Kallscheuer et al. 2020b), as well as in environments contaminated with metals (Akob et al. 2007; Halter et al. 2011) or hydrocarbons (Abed et al. 2011). Although widely distributed in the environment and abundant in some habitats, the number of isolated species is still moderate due to the difficulty of obtaining axenic cultures, mainly because they usually are slow growing bacteria with doubling times reported from around 5 h to up to 140 h (Vitorino et al. 2021; Vitorino and Lage 2022; Wiegand et al. 2020b). Recent efforts to expand the current collection of planctomycetal axenic cultures have led to the rapid increase of the description of new genera and species (Vitorino and Lage 2022; Wiegand et al. 2020b). An example is the newly described genus *Stieleria* (Kallscheuer et al. 2020a; Sandargo et al. 2020; Surup et al. 2020), which currently comprises four described species, to which the production of secondary metabolites with antimicrobial activity as well as potential quorum-sensing mechanisms have been linked (Kallscheuer et al. 2020a; Sandargo et al. 2020; Vitorino et al. 2022). *Stieleria maiorica* Mal15^T^, the first described strain within the genus, was isolated from a seawater sediment sample from Mallorca island, Spain (Kallscheuer et al. 2020a). *Stieleria neptunia* Enr13^T^ was isolated from leaves of *Posidonia* sp. collected close to Panarea island, Italy (Sandargo et al. 2020), and *Stieleria varia* (type strain Pla52n^T^), was isolated from the biofilms on wood particles incubated in the Baltic Sea (Surup et al. 2020). More recently, *Stieleria sedimenti* ICT_E10.1^T^ was isolated from sediments retrieved from the Tagus river estuary in Portugal (Vitorino et al. 2022). A taxonomic conflict has been previously detected between the genus Stieleria and the genus “Roseiconus”, which has two described species “Roseiconus lacunae” and “Roseiconus nitratireducens” (Kumar et al. 2021; Vitorino and Lage 2022). Although these two species do not have validly published names, their descriptions have been published and as such are included in this analysis. Here, we describe the strain TO1_6^T^ (= CECT 30432^T^, = LMG 32465^T^), which was isolated from a river water sample from Tagus river, Portugal, for which the name *Stieleria tagensis* sp. nov. is proposed.

## Materials and methods

### Isolation and cultivation

Isolate TO1_6^T^ was retrieved from a water sample from Tagus river, Alcochete (38° 45′ 20″ N 8° 57′ 55″ W) in May 2021. At the time of sampling, the water temperature and salinity (% (w/v) NaCl) were 20 °C and 1.54%, respectively. In brief, 250 mL of river water were filtered through a 0.22 µm pore size Whatman sterile membrane filter, which was then placed on M13 + NAG medium (0.25 g/L peptone, 0.25 g/L yeast extract, 50 mL/L 0.1 mM Tris–HCl buffer (pH 7.5), 10 mL/L 2.5% (w/v) glucose solution, 10 mL/L 5% (w/v) N-acetylglucosamine (NAG) solution, 10 mL vitamin solution (Lage and Bondoso 2011), 20 mL Hutner’s Basal Salts solution (Cohen-Bazire et al. 1957), 90% (v/v) of natural sea water, 1.6 g/L of agar) supplemented with streptomycin (1000 µg/mL), vancomycin (40 µg/mL) and cycloheximide (20 µg/mL). The membrane was incubated at 26 °C and checked routinely for colonies for almost a month, when a small pink colony was retrieved and labeled as strain TO1_6. Strain TO1_6 was maintained on M13 + NAG medium at 26 °C and preserved in medium M13 + NAG supplemented with 20% (v/v) glycerol at − 80 °C.

### Phylogenetic inference and genome analysis

DNA extraction of an axenic culture of strain TO1_6^T^ was performed using the E.Z.N.A. Bacterial DNA Isolation Kit (Omega BioTek) according to the manufacturer’s instructions. Extracted genomic DNA was used for PCR amplification of the 16S rRNA gene using the universal primers 27F and 1492R (Lane 1991) and for genome sequencing. The PCR mixture of 25 µL was prepared with 12.5 µL NZYTaq 2 × Green Master Mix (NZYTech), 0.25 µL of primer 27F (10 mM), 0.25 µL of primer 1492R (10 mM), 10 µL of H_2_O and 2 µL of DNA. The PCR was performed in a MyCycler™ Thermo Cycler (Bio-Rad) according to the following steps: initial denaturation at 95 °C for 5 min; 30 cycles of 95 °C for 1 min, 56 °C for 1 min, and 72 °C for 1:30 min; and last step of final extension at 72 °C for 10 min. The PCR products were then visualized after electrophoresis on a 0.8% (w/v) agarose gel in 1 × Tris–Acetate-EDTA (TAE) buffer stained with GreenSafe Premium (NZYTech). All amplicons were then purified with a GFX PCR DNA and Gel Band Purification Kit (Cytiva) and sent for sequencing at Eurofins Genomics. The obtained sequences were trimmed and analyzed using Geneious Prime 2021, and the consensus sequence was compared with the National Center for Biotechnology Information (NCBI) Genbank database (Benson et al. 2013) using NCBI’s Standard Nucleotide BLAST search (Altschul et al. 1990; Johnson et al. 2008) and with the 16S rRNA-based ID tool from the EzBioCloud platform (Yoon et al. 2017a) for phylogenetic affiliation.

Evolutionary analyses were carried out using MEGA7 software (Kumar et al. 2016) using 16S rRNA gene sequences retrieved from GenBank (Benson et al. 2013) from closely related strains and the type strain of *Phycisphaera mikurensis*, which was used as the outgroup, and aligned using ClustalW (Thompson et al. 1994). The dendrogram was constructed with the maximum likelihood method based on the General Time Reversible model and gamma distribution with invariant sites (G + I) (Tamura and Nei 1993) and phylogeny was tested with the bootstrap method with 1000 replications.

For genome sequencing, the library preparation was performed using the DNA Prep kit (Illumina), followed by sequencing on a MiSeq system (Illumina). The de novo genome assembly was performed using CLC Genomics Workbench (QIAGEN) version 21.0.1 and the completeness and contamination of the assembled genome was analyzed by checkM version 1.20 (Parks et al. 2015). Open reading frame calling was performed using Prodigal version 2.6.3 (Hyatt et al. 2010) and the coding sequences were annotated with Prokka version 1.14.6 (Seemann 2014). Comparative genomic analyses between strain TO1_6^T^ and its current closely related species were performed. Genomes of closely related type strains were obtained from the NCBI Genbank database (Benson et al. 2013) and annotated simultaneously with the TO1_6^T^ genome. The ANI values were calculated using CJ Bioscience's online ANI calculator at the EzBioCloud platform (Yoon et al. 2017a, b). The AAI values were calculated using the enveomics collection online ANI/AAI-Matrix: All-vs-all ANI/AAI matrix calculator (Rodriguez-R and Konstantinidis 2014, 2016). The *rpoB* and 16S rRNA genes identities were calculated with NCBI’s Standard Nucleotide BLAST search (Altschul et al. 1990; Johnson et al. 2008). The full *rpoB* gene enconding sequences were retrieved from genome annotation. The POCP was determined as previously described (Qin et al. 2014).

For the multi-locus sequence analysis (MLSA)-based phylogenetic tree, autoMLST was used. The analysis was performed with the Denovo method in fast alignment mode (MAFFT FFT-NS-2) with the default set of MLST genes and with filtering of inconsistent MLST genes and IQ-TREE Ultrafast Bootstrap analysis with 1000 replicates (Alanjary et al. 2019). Visualization of the tree was performed on the iTOL platform (Letunic and Bork 2019).

The presence of putative biosynthetic gene clusters was analyzed using antiSMASH 6.0 with relaxed detection strictness and with all extra features activated (Blin et al. 2021). The presence of antibiotic resistance genes was assessed with the CARD-RGI platform, using the genomic DNA sequence as input, considering only perfect and strict hits and excluding the nudge (Alcock et al. 2020).

### Morphological and physiological characterization

The morphological characterization of strain TO1_6^T^ was performed using optical and transmission electron microscopy (OM, TEM). Cell preparation for TEM followed a previously described protocol (Godinho et al. 2021) with slight modifications. Briefly, cells were harvested after 4 days of cultivation and were fixed in 2.5% (v/v) glutaraldehyde in marine buffer (Watson et al. 1986) for 2 h. Next, the cells were post-fixed in 1% (w/v) osmium tetroxide for 4 h in marine buffer and subsequently with 1% (w/v) uranyl acetate for 1 h. Dehydration was carried out with a graded ethanol series, followed by incubation in propylene oxide and embedding in Epon resin. Ultrathin sections of the embedded material were stained first with uranyl acetate, and then with Reynolds lead citrate, for 10 min each. Samples were visualized in a JEOL 100CXII transmission electron microscope.

All physiological tests were carried out in triplicates in a volume of 10 mL of M13 + NAG medium at room temperature for 4 days, after which the final OD_600nm_ was measured (Thermo Scientific™ GENESYS™ 10UV Spectrophotometer), unless otherwise stated. The range and optimal temperatures were determined on M13 + NAG agar medium plates incubated from 5 to 40 °C, with 5 °C increments, by placing 3 droplets of 10 µL of exponential phase culture on each plate and incubating for 7 days at the respective testing temperature. Growth was observed visually. Salinity was tested in M13 + NAG broth prepared with artificial seawater (ASW) (Harrison et al. 1980) without NaCl and subsequently supplemented with different concentrations of NaCl from 0 to 12% (w/v) in 1% increments. For the determination of optimum and growth range regarding pH, M13 + NAG broth with pH ranging from 5.0 to 10.0, at 0.5 units intervals, was prepared using the following buffer systems: citrate buffer 0.1 M for pH 5.0, MES 0.1 M for pH 5.5–6.5, Tris–HCl 1 M for pH 7.0–8.5 and CHES 1 M for pH 9.0–10.0. To assess vitamin requirements, M13 + NAG broth without vitamins solution no. 6 (Lage and Bondoso 2011) was used, and for each experiment one of the following vitamins was individually added: biotin (20 µg/L), folic acid (20 µg/L), riboflavin (50 µg/L), thiamine-HCl (50 µg/L), nicotinamide (100 µg/L), calcium d-pantothenate (50 µg/L) and vitamin B_12_ (1 µg/L). For nitrogen utilization assays, the base of M13 + NAG broth without peptone, yeast extract and NAG was used and later individually supplemented with a total of 15 different nitrogen sources at 0.1% (w/v), namely l-arginine, l-tyrosine, l-threonine, l-glutamine, l-cysteine, l-methionine, l-isoleucine, l-serine, l-aspartic acid, peptone, yeast extract, NAG, sodium nitrate, sodium nitrite and urea. A negative control without any nitrogen source was included, as well as a positive control using standard M13 + NAG broth. These cultures were incubated for 4 days before measuring the results by absorbance reading at 600 nm. For carbon utilization assays, M13 + NAG broth without peptone and NAG, but with 1 g/L of yeast extract was used. A total of 16 different carbon sources were then added individually at 0.1% (w/v), namely d-arabinose, cellobiose, dulcitol, d-galactose, glycerol, *myo*-inositol, lactose, maltose, d-mannitol, d-sorbitol, saccharose, trehalose, d-xylose, d-glucose, raffinose and dextran. A negative control without any carbon source was included and M13 + NAG broth was applied as positive control. These cultures were incubated for 7 days before measuring the results by absorbance reading at 600 nm.

The growth rate of strain TO1_6 was inferred in medium M13 + NAG at 25 °C and 1000 rpm in a BioSan™ RTS-1C Personal bioreactor. Anaerobic and microaerophilic growth were tested using the GENbox system (bioMérieux S.A., France) containing a generator sachet, either Genbox anaer or Genbox microaer (bioMérieux S.A.), in which plates of M13 + NAG agar medium inoculated with strain TO1_6^T^ were incubated at 26 °C for 1 month. Antibiotic susceptibility was evaluated by the modified Kirby-Bauer method as previously described (Godinho et al. 2019) in M13 medium. The tested antibiotics (amount per disc in brackets) were amikacin (30 µg), gentamicin (10 µg), tobramycin (10 µg), kanamycin (30 µg), chloramphenicol (30 µg), amoxicillin (10 µg), amoxicillin-clavulanic acid (30 µg), aztreonam (30 µg), cefotaxime (30 µg), cefoxitin (30 µg), ceftazidime (30 µg), imipenem (10 µg), meropenem (10 µg), piperacillin (100 µg), piperacillin-tazobactam (110 µg), fosfomycin (50 µg), teicoplanin (30 µg), vancomycin (30 µg), clindamycin (2 µg), erythromycin (15 µg), nitrofurantoin (300 µg), colistin sulphate (10 µg), polymyxin B (300 IU), ciprofloxacin (5 µg), nalidixic acid (30 µg), doxycycline (30 µg) and tetracycline (30 µg).

The fatty acid content of *Stieleria tagensis* T01_6^T^ and *Stieleria sedimenti* ICT_E10.1^T^ was evaluated by gas chromatography (GC) using the MIDI’s Sherlock™ Microbial ID System. Strains were first cultured for 4 days at 25 °C in modified R2A medium plates prepared as followed (per liter of Milli-Q water): 18.2 g of Difco™ R2A Agar powder and 30 g of Instant Ocean® Sea Salt. After autoclaving, the following supplements were added by filtration (0.22 µm pore filter): 10 mL vitamin solution (Lage and Bondoso 2011), 20 mL Hutner’s basal salts solution (Cohen-Bazire et al. 1957) and 40 mL glucose solution (stock at 2.5% w/v). Biomass was collected and the fatty acids obtained by saponification, methylation and extraction following the manufacturer’s instructions (MIDI 2017; Sasser 1990). Finally, the fatty acid content was analyzed in an Agilent 6890N Network Gas Chromatograph equipment.

## Results and discussion

### 16S rRNA gene sequencing and phylogenetic analysis

A nucleotide BLAST search was conducted using the partial (1292 bp) 16S rRNA gene sequence of strain TO1_6^T^ as query (in August 2022), and all the hits with more than 98.65% identity (the proposed threshold for species delineation) were analyzed. A total of five distinct hits were obtained from which three are from isolates and two from metagenomic samples. The closest hits are from metagenomic samples from the macroalga *Chondrus crispus* collected in Foz, Porto, North of Portugal in summer, and from seawater next to dolphin I in San Diego, California, USA, with 98.75 and 98.73% identities, respectively. The three isolates are strains TBK1, Enr13^T^ and JC639, all with 98.68% identity. TBK1 was isolated from iron hydroxide deposits in Valu Fa ridge, Pacific Ocean (Storesund et al. 2018), while strain Enr13^T^ was isolated from leaves of *Posidonia* sp. collected close to the Panarea island, Italy. Strain JC639 was isolated in Tamil Nadu, India, but no information regarding sample type was provided. This indicates a disperse geographic distribution of members of the genus *Stieleria*.

**Fig. 1:**
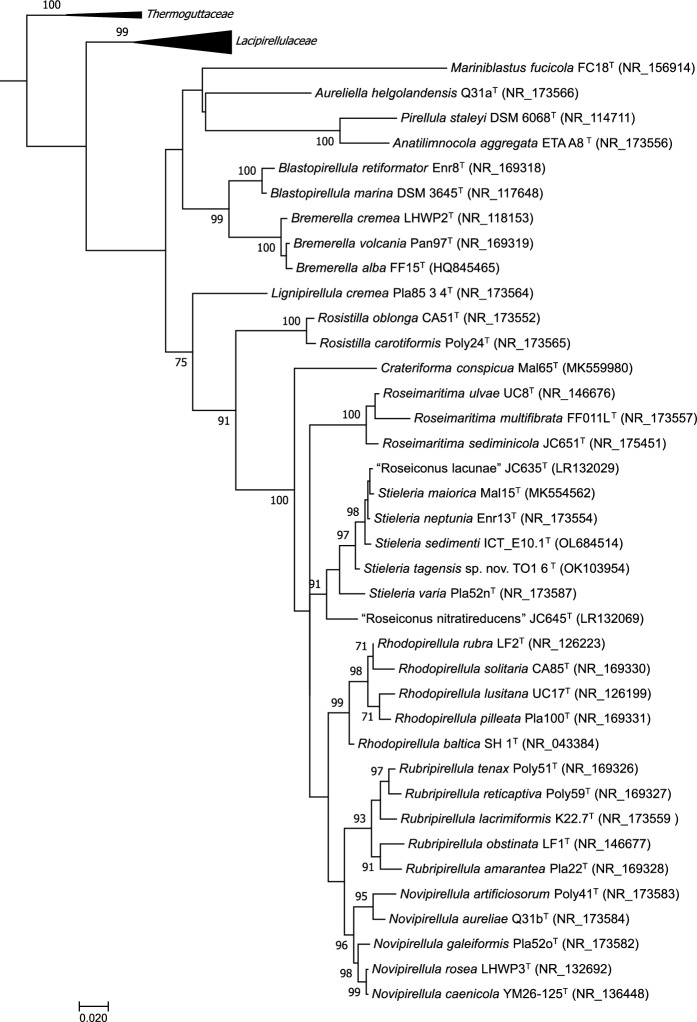
Phylogenetic 16S rRNA gene sequence-based dendrogram demonstrating the relationship between the type species of the different genera within the families *Lacipirellulaceae*, *Thermoguttaceae* and *Pirellulaceae* and the described species of the genus *Stieleria*, including the new strain TO1_6^T^. *Phycisphaera mikurensis* was used as outgroup. The numbers on the branches refer to percentage of trees in which the associated taxa clustered together from the total of bootstrap replications. GenBank accession numbers are presented. The scale bar refers to 0.02 substitutions per nucleotide position

According to the nucleotide BLAST and the EzBioCloud analysis of the 16S rRNA gene of strain TO1_6^T^ the closest sequence belongs to the type strain of the species *Stieleria neptunia,* strain Enr13^T^, with 98.68% identity. Strain TO1_6^T^ clusters with the described species of the genus *Stieleria* in maximum likelihood phylogenetic trees based on 16S rRNA gene sequences (Fig. 1). The 16S rRNA gene similarity is presented in Table 1 taking into consideration described species of the genera *Stieleria* and “Roseiconus” and strain TO1_6^T^. The above-mentioned 16S rRNA gene similarity of strain TO1_6^T^ to *S. neptunia* strain Enr13^T^ is slightly above the 98.65% threshold for species delimitation (Kim et al. 2014). However, the 16S rRNA gene similarity alone has often been reported as insufficient to delineate species within the phylum *Planctomycetota*, as for example in case of strains that share 99.01% similarity, like *S. neptunia* strain Enr13^T^ and *S. maiorica* Mal15^T^. Even higher 16S rRNA gene sequence similarities have been observed for strains within the phylum that turned out to belong to separate species based on whole-genome-based phylogenetic analyses (Kallscheuer et al. 2020a; Kohn et al. 2020; Sandargo et al. 2020; Wiegand et al. 2020a). Additional phylogenetic markers such as the sequence of the *rpoB* gene (encoding the β-subunit of the RNA polymerase), ANI, AAI and POCP are commonly used for the taxonomic delimitation within this phylum.

**Table 1:** Phylogenetic marker values (in %) between the different described species of the genus *Stieleria* and isolate TO1_6^T^

		TO1_6^T^	Pla52n^T^	Enr13^T^	Mal15^T^	ICT_E10.1^T^	JC635^T^
ANI	*S. varia* Pla52n^T^	70.98					
	*S. neptunia* Enr13^T^	73.82	71.40				
	*S. maiorica* Mal15^T^	73.63	71.43	80.13			
	*S. sedimenti* ICT_E10.1^T^	73.67	71.39	88.54	79.85		
	“R. lacunae” JC635^T^	71.18	69.88	71.68	72.26	71.51	
	“R. nitratireducens” JC645^T^	72.57	70.86	73.05	73.23	73.00	71.05
rpoB	*S. varia* Pla52n^T^	84.27					
	*S. neptunia* Enr13^T^	88.45	84.40				
	*S. maiorica* Mal15^T^	88.75	85.00	91.19			
	*S. sedimenti* ICT_E10.1^T^	88.65	84.16	94.01	90.91		
	“R. lacunae” JC635^T^	84.39	82.17	84.76	85.92	84.97	
	“R. nitratireducens” JC645^T^	86.36	83.91	86.86	87.82	86.86	83.84
AAI	*S. varia* Pla52n^T^	61.28					
	*S. neptunia* Enr13^T^	69.16	60.80				
	*S. maiorica* Mal15^T^	69.03	61.03	80.70			
	*S. sedimenti* ICT_E10.1^T^	69.48	61.70	89.51	81.21		
	“R. lacunae” JC635^T^	64.92	59.56	65.17	65.75	65.37	
	“R. nitratireducens” JC645^T^	66.93	61.19	65.99	66.45	66.61	63.92
POCP	*S. varia* Pla52n^T^	56.79					
	*S. neptunia* Enr13^T^	63.20	56.54				
	*S. maiorica* Mal15^T^	66.00	57.59	76.31			
	*S. sedimenti* ICT_E10.1^T^	64.21	58.69	80.04	77.96		
	“R. lacunae” JC635^T^	62.45	55.24	60.92	65.55	62.38	
	“R. nitratireducens” JC645^T^	65.57	58.63	60.63	63.88	63.29	61.76
16S rRNA gene	*S. varia* Pla52n^T^	97.14					
	*S. neptunia* Enr13^T^	98.68	96.19				
	*S. maiorica* Mal15^T^	98.45	96.27	99.01			
	*S. sedimenti* ICT_E10.1^T^	97.97	95.95	98.75	98.75		
	“R. lacunae” JC635^T^	98.53	95.77	99.36	99.15	98.59	
	“R. nitratireducens” JC645^T^	96.20	95.41	96.68	96.90	95.90	96.83

### Genome analysis

The main features of the genome of strain TO1_6^T^ are presented in Table 2. Genome sequencing revealed a genome size of 7.77 Mbp and DNA G + C content of 56.3%. The assembly resulted in 196 contigs and the annotation resulted in 5752 protein coding sequences, 3803 of which are annotated as hypothetical proteins, representing a total of 66% of hypothetical proteins. The genome also encodes 11 giant genes (*i.e.* genes > 10 kb), most of which code for proteins with unknown function, with the exception of one that was automatically annotated as putative 6-phosphogluconolactonase. When compared to the other species of the genus *Stieleria*, the genome of strain TO1_6^T^ has a DNA G + C content closer to *S. varia* than to the other two species, but it has a smaller size when compared to the genomes of the other three described *Stieleria* species and has a higher coding density. Remarkably, even though the genome is smaller, it harbors a higher percentage of hypothetical proteins (66%), which exceeds the range of 40–50% hypothetical proteins typically found in the genomes of other members of the phylum *Planctomycetota* (Wiegand et al. 2020b). The complete 16S rRNA gene (1529 bp) was retrieved from the annotation of the genome and it had 100% similarity with the partial 16S rRNA gene obtained from amplicon sequencing.

**Table 2:** Comparison of genomic features between isolate TO1_6^T^ and the described species of the genus *Stieleria*

Features	TO1_6^T^	Pla52n^T^	Enr13^T^	Mal15^T^	ICT_E10.1^ T^	*JC635*^*T*^	*JC645*^*T*^
Genome size	7,773,954	9,586,696*	10,975,817^a^	9,894,293^b^	9,813,311^c^	7,951,142^d^	8,196,902^d^
DNA G + C content (%)	56.3	56.0*	58.9^a^	59.3^b^	58.8^c^	55.1^d^	60.0^d^
Completeness	99.93	98.28*	98.28^a^	98.28^b^	99.93^c^	N.D	N.D
Contamination	0	3.45*	1.72^a^	2.59^b^	0	N.D	N.D
Total genes	5802	7094*	7904^a^	7016^b^	N.D	5570^d^	5806^d^
Predicted protein-coding genes	5752	6998*	7797^a^	6920^b^	6964^c^	5410^d^	5637^d^
Predicted hypothetical proteins	3803	3223*	3425^a^	2897^b^	4578^c^	N.D	N.D
Hypothetical proteins (%)	65.55	45.43	43.33	41.29	66^c^	N.D	N.D
Coding density	89.03	87.22*	85.98^a^	86.95^b^	N.D	N.D	N.D
tRNA genes	46	80*	99^a^	81^b^	109^c^	72^d^	74^d^

*Data from Surup et al. (2020)

^a^Data from Sandargo et al. (2020)

^b^Data from Kallscheuer et al. (2020a)

^c^Data from Vitorino et al. (2022)

^d^Data from Kumar et al. (2021)

Given that members of the genus *Stieleria* have been linked to the production of bioactive compounds (Kallscheuer et al. 2020a; Sandargo et al. 2020), prediction of biosynthetic gene clusters potentially associated to secondary metabolite production was performed with antiSMASH. The analysis yielded a total of seven clusters, two of which are putatively related to the production of terpenes, while the remaining are related to the biosynthesis of polyketides (one type I and one type III polyketide synthases), non-ribosomal peptides (one cluster), hybrid polyketides/non-ribosomal peptides (one cluster) and *N*-acyl amino acids (one cluster) (Supplementary Table 1). The predicted terpene biosynthetic enzymes seem to be related to the production of carotenoids and the production of antimicrobial compounds. Antibiotic activity is also predicted regarding the clusters of both polyketide synthases classes and of the non-ribosomal peptide. The PKS/NRPS hybrid cluster might be related to the production of antioxidants. The type strains of two of the three characterized *Stieleria* species have been shown to produce stieleriacines. The key reactions of their biosynthesis were proposed to be catalyzed by enzymes encoded in an *N*-acyl amino acid cluster. However, when comparing the putative *N*-Acyl amino acid cluster identified in strain TO1_6^T^ with the one described in *S. neptunia* Enr13^T^, only 35% of the genes from TO1_6^T^ cluster show similarity to the ones in Enr13^T^, suggesting that the two clusters, although predicted to belong to the same class, may differ. This is not unexpected when taking into account that *S. maiorica* Mal15^T^ and *S. neptunia* Enr13^T^ produce compounds that belong to the same class (namely stieleriacines), but produce compounds that chemically differ in fatty acid chain length and aromatic ring substitutions (Kallscheuer et al. 2020a; Sandargo et al. 2020). Apart from this, the cluster in *S. maiorica* includes several additional genes coding for putative transporters and cell wall biosynthesis proteins, that are not necessarily related to stieleriacine biosynthesis.

**Fig. 2:**
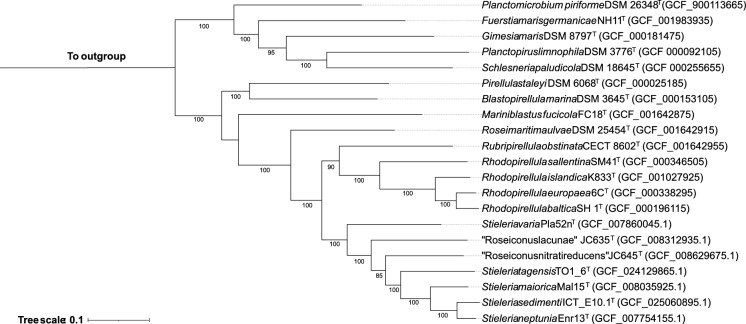
Multi-locus sequence analysis-based phylogenetic tree highlighting the position of the novel strain TO1_6^T^. The web-based tool autoMLST was used to construct the phylogenetic tree, with 1000 bootstrap (values shown in % at the nodes). RefSeq accession numbers are presented. Genomes from *Verrucomicrobium spinosum* DSM 4136^T^ and *Phycisphaera mikurensis* NBRC 102666^T^ were used as outgroups. Scale bar represents 0.10 substitutions per nucleotide position

Additional phylogenetic markers such as similarity of *rpoB* gene sequence, ANI, AAI and POCP are commonly used for the purpose of species and genus delineation within this phylum. The results of these different markers are reported in Table 1. POCP values show that TO1_6^T^ belongs to the genus *Stieleria* (values above 50% with the three described species (Qin et al. 2014)), while ANI, AAI and *rpoB* similarity reinforce that strain TO1_6^T^ represents a new species within this genus. The obtained values of 70.98–73.82% are below the 95% threshold for ANI (Goris et al. 2007), 61.28–69.16% are below the 95–98% threshold for AAI (Konstantinidis and Tiedje 2005), and *rpoB* identities are between 82.34% and 86.55% which are also below the established 96.3% threshold (Bondoso et al. 2013). Strain TO1_6^T^ clusters with the described species of the genus *Stieleria* in maximum likelihood phylogenetic trees based on multi-locus sequence analysis but on a separate branch (Fig. 2).

### Morphology and physiology

**Fig. 3:**
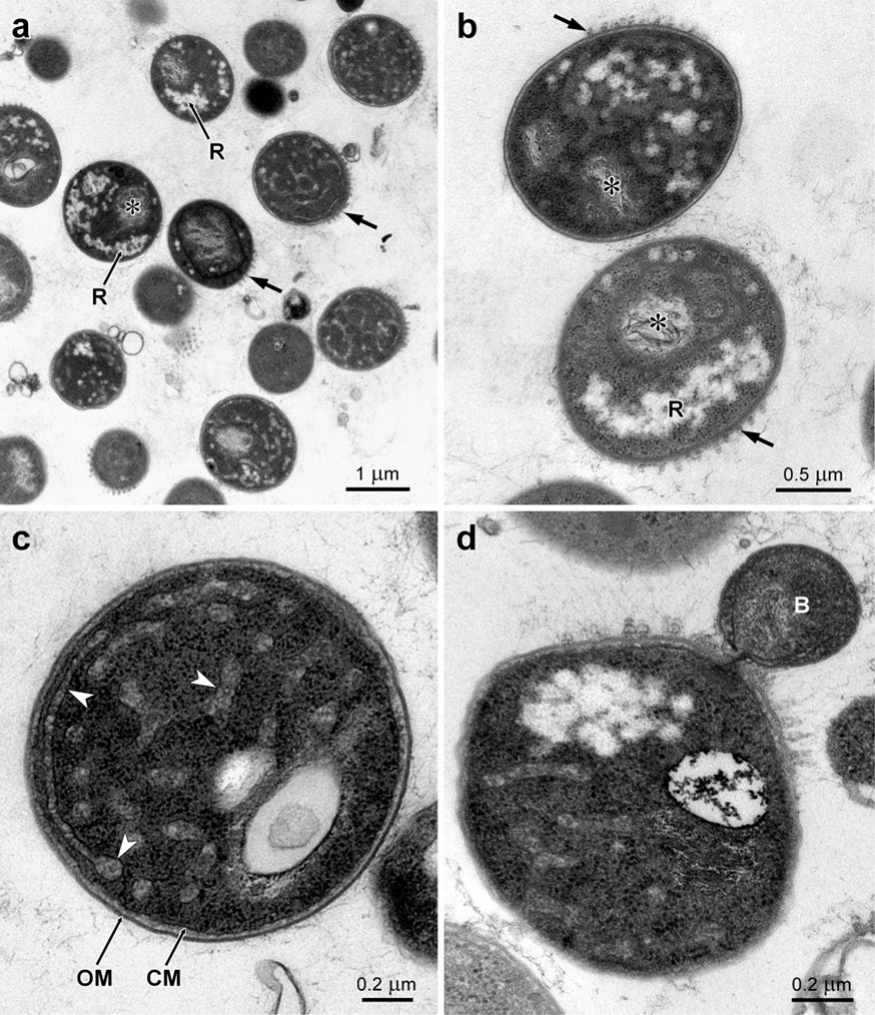
TEM images of strain TO1_6^T^ depicting its ultrastructure. R—inclusion storage bodies; asterisk—Condensed DNA; OM—outer membrane; CM—cytoplasmic membrane; arrow head—invaginations of the cytoplasmic membrane; arrow—fimbriae; B—bud

When cultivated on M13 + NAG agar, isolate TO1_6^T^ forms small, circular and white/beige colonies in the initial phases of incubation, but eventually these become light pink after a couple of days. When grown in M13 + NAG broth, cells of strain TO1_6^T^ show the same pattern of pigmentation but grow aggregated in flakes, with fresh cultures having a clear medium with flakes. Turbidity is only observed in old cultures. Cells in the aggregates can be separated by vortexing. Under the OM it was possible to visualize the presence of rosettes, as well as single cells, and the occurrence of budding. The cells of strain TO1_6^T^ are typically pear-shaped, but larger and more round-shaped cells can also occur. TEM micrographs showed the typical characteristics of the ultrastructure of members of the phylum *Planctomycetota* (Fig. 3). In detail, we observed complex membrane invaginations, condensed DNA, ribosomes as well as inclusion storage bodies. Thick polar fimbriae are present.

Strain TO1_6^T^ was able to grow between 15 and 30 °C, with optimal growth at 20–25 °C, and in the presence of 0–11% (w/v) NaCl, with optimum growth at 1%. Growth was observed over a pH ranging from 5.0 to 9.5, with optimal growth at pH 7.0–7.5. Strain TO1_6^T^ did not show an altered growth behavior in the absence of any of the tested vitamins, except for vitamin B_12_ and thiamine-HCl which promoted its growth when used to supplement M13 + NAG medium. Regarding nitrogen source utilization, strain TO1_6^T^ was able to use l-arginine, l-tyrosine, l-threonine, l-glutamine, l-isoleucine, l-aspartic acid, peptone, yeast extract, NAG and nitrate. Optimal growth was obtained with yeast extract. For carbon source utilization, strain TO1_6^T^ was able to use d-arabinose, cellobiose, glycerol, *myo*-inositol, lactose, maltose, d-mannitol, d-sorbitol, saccharose, trehalose, d-xylose, d-glucose, raffinose and dextran. Optimal growth was obtained with dextran as carbon source. A doubling time of 17.2 h was observed in M13 + NAG medium. Strain TO1_6^T^ was able to grow under microaerophilic conditions but not under anaerobic conditions. The fatty acid content of *S. tagensis* TO1_6^T^ and *S. sedimenti* ICT_E10.1^T^ are presented in Supplementary Table 2. Major fatty acids for both strains are C_16:0_ and C_18:1_ω9c.

A comparison between the morphological and physiological characteristics between isolate TO1_6^T^ and other described species of the genus *Stieleria* is presented in Table 3. Isolate TO1_6^T^ differs from other members of the genus by its initial white to beige pigmentation, longer generation time, and lower optimal temperature.

**Table 3:** Morphological and physiological characteristics of isolate TO1_6^T^ in comparison with the described species of the genus *Stieleria*

Features	TO1_6^T^	Pla52n^T^	Enr13^T^	Mal15^T^	ICT_E10.1^ T^	JC635^T^	JC645^T^
Cell size (µm)	1.45 ± 0.1 × 1.02 ± 0.2	1.8 ± 0.3 × 0.9 ± 0.2*	1.6 ± 0.1 × 1.1 ± 0.1^b^	1.9 ± 0.2 × 1.4 ± 0.2^c^	1.7 ± 0.3 × 1.4 ± 0.3^d^	1.2–1.5 × 0.5–0.9^e^	1.2–1.5 × 0.5–0.9^e^
Shape	Round to pear-shaped	Ovoid to round grain rice-shaped*	Round grain rice-shaped^b^	Round to pear-shaped^c^	Spherical to ovoid^d^	Cone to pear^e^	Spherical to ovoid^e^
Colony colour	White to light pink	Light orange pigmentation*	Pink-coloured^b^	Pink-coloured^c^	Pink^d^	Light pink^e^	Pink^e^
Rosette formation	Yes	Yes*	Yes^b^	Yes^c^	No^d^	N.D	N.D
Cell division	Budding	Polar budding*	Polar budding^b^	Polar budding^c^	Budding^d^	Polar budding^e^	Polar budding^e^
Source	Brackish water from Tagus River	Wood particles incubated in marine/brackish conditions*^a^	Leaves of the marine plant *Posidonia* sp.^b^	Marine sediments^c^	Brackish sediments^d^	Lagoon sediments^e^	Lagoon sediments^e^
Crateriform structures	Yes	Yes*	Yes^b^	Yes^c^	No^d^	Yes^e^	Yes^e^
Doubling time	17.2 h	11 h*	13 h^b^	7.5 h^c^	N.D	N.D	N.D
*Temperature for growth*							
Optimum	20–25 °C	33 °C*	28 °C^b^	35 °C^c^	25^d^	30^e^	30^e^
Range	15–30 °C	15–36 °C*	9–35 °C^b^	11–< 37 °C ^c^	20–30^d^	10–35^e^	10–35^e^
*pH for growth*							
Optimum	7.0 – 7.5	7.5*	7.5^b^	7.5^c^	N.D	8.0^e^	8.0^e^
Range	5.0–9.5	6.0–8.0*	6.5–9.0^b^	5.5–9.0 ^c^	6.5–11^d^	6.0–9.0^e^	7.0–9.0^e^
*NaCl for growth*							
Optimum	0.01	N.D	N.D	N.D	N.D	0.03^e^	0.04^e^
Range	0–5%	N.D	N.D	N.D	0.5–3%^d^	1–5%^e^	1–7%^e^
Carbon sources	d-arabinose, cellobiose, glycerol, *myo*-inositol, lactose, maltose, d-manitol, d-sorbitol, saccharose, trehalose, d-xylose, d-glucose, raffinose and dextran	N.D	N.D	NAG, arabinose, cellobiose, fucose, fructose, galactose, gentiobiose, glucose, gluconic acid, glucuronamide, glucuronic acid, lactose, lactulose, mannose, melibiose, glucoside, draffinose, rhamnose, sucrose, trehalose, turanose, psicose^c^	NAG, cellobiose, galactose, fructose, lactose, arabinose, xylose and glucose^d^	d-glucose, fructose, mannose, maltose, sucrose, starch, d-xylose, lactose, d-galactose and rhamnose^e^	d-glucose, fructose, mannose, maltose, sucrose, starch, d-xylose, rhamnose, pyruvate and inulin
Nitrogen sources	l-arginine, l-tyrosine, l-threonine, l-glutamine, l-isoleucine, l-aspartic acid, peptone, yeast extract, NAG and nitrate	N.D	N.D	N.D	NAG, peptone, yeast extract, ammonium sulfate, casamino acids, urea, sodium nitrate, asparagine, glutamine, histidine, phenylalanine, tryptophan, and alanine^d^	Ammonium sulphate, peptone, l-serine, DL-threonine, l-leucine and DL-alanine, l-isoleucine, l-phenylalanine, l-glutamic acid and l-aspartic acid^e^	Ammonium sulphate, peptone, l-serine, dlthreonine, l-leucine, dl-alanine, cysteine, l-glutamine, l-proline and urea^e^
*Fatty acids*							
C_16:0_ (%)	31.33	N.D	N.D	N.D	31.75	16.8^e^	17.4^e^
C_18:1_ω9c (%)	43.68	N.D	N.D	N.D	46.94	21.4^e^	29.5^e^

*Data from (Surup et al. 2020)

^a^Data from (Oberbeckmann et al. 2017)

^b^Data from (Sandargo et al. 2020)

^c^Data from (Kallscheuer et al. 2020a)

^d^Data from (Vitorino et al. 2022)

^e^Data from (Kumar et al. 2021)

The results from the antibiotic susceptibility testing are presented in Table 4. Strain TO1_6^T^ showed no susceptibility to any of the tested antibiotics that target cell wall biosynthesis, including the two tested β-lactam/β-lactamase inhibitor combinations. Resistance to glycopeptides such as vancomycin and teicoplanin was expected since these antibiotic molecules are big and usually unable to cross the outer membrane of Gram-negative bacteria (Blair et al. 2015). The resistance to β-lactams, β-lactams/β-lactamase inhibitor combinations and fosfomycin, is in agreement with previous reports for this phylum (Cayrou et al. 2010; Godinho et al. 2019; König et al. 1984). Although some exceptions have been previously reported (Hu et al. 2013; Ivanova et al. 2021; Zaicnikova et al. 2011), the vast majority of the phylum members, for which antibiotic susceptibility has been tested, showed high resistance to various antibiotics that target cell wall biosynthesis (Cayrou et al. 2010; Godinho et al. 2019; Ivanova et al. 2021; König et al. 1984; Vitorino and Lage 2022). Regarding the remaining groups of antibiotics, strain TO1_6^T^ showed mixed results. For compounds that target protein biosynthesis, the following observations were made: the strain was resistant to the four tested aminoglycosides, but was susceptible to chloramphenicol, clindamycin and erythromycin, and showed small inhibition zone diameters for doxycycline and tetracycline. Susceptibility to erythromycin has been reported for this phylum (Cayrou et al. 2010; Godinho et al. 2019; Ivanova et al. 2021). For those that target DNA replication, strain TO1_6^T^ showed resistance to nalidixic acid, but was susceptible to ciprofloxacin. It was also susceptible to nitrofurantoin. And finally, among the ones that target the structure and integrity of the cell membrane, it showed resistance to colistin and susceptibility to polymyxin B. Given the broad range of resistance to antibiotics that target the cell wall biosynthesis, as well as to aminoglycosides, it was hypothesized that antibiotic resistance genes should be present in the genome of strain TO1_6^T^. However, prediction of antibiotic resistance genes with CARD-RGI online platform yielded only 3 hits, all of which are for *adeF*. According to the CARD, *adeF* codes for the membrane fusion protein of the multidrug efflux complex AdeFGH which has been linked to the resistance to fluoroquinolones, tetracycline, tigecycline, chloramphenicol, clindamycin, trimethoprim, and sulfamethoxazole (Alcock et al. 2020; Coyne et al. 2010). Even though this efflux pump might be responsible for resistance to nalidixic acid or tetracycline, no gene hits for the rest of the components of this pump were retrieved and other mechanisms could be at play. Surprisingly, no currently known genetic determinants of resistance to either β-lactams, fosfomycin or aminoglycosides were found.

**Table 4:** Antibiotic susceptibility profile obtained for strain TO1_6^T^

Class	Target	Antibiotics	TO1_6^T^
Aminoglycosides	Protein biosynthesis	Amikacin	R
Aminoglycosides	Protein biosynthesis	Gentamicin	R
Aminoglycosides	Protein biosynthesis	Kanamycin	R
Aminoglycosides	Protein biosynthesis	Tobramycin	R
Amphenicol	Protein biosynthesis	Chloramphenicol	50
Beta-lactams	Cell wall biosynthesis	Amoxycillin	R
Beta-lactams	Cell wall biosynthesis	Amoxycillin/Clavulanic acid	R
Beta-lactams	Cell wall biosynthesis	Aztreonam	R
Beta-lactams	Cell wall biosynthesis	Cefepime	R
Beta-lactams	Cell wall biosynthesis	Cefotaxime	R
Beta-lactams	Cell wall biosynthesis	Cefoxitin	R
Beta-lactams	Cell wall biosynthesis	Ceftazidime	R
Beta-lactams	Cell wall biosynthesis	Imipenem	R
Beta-lactams	Cell wall biosynthesis	Meropenem	R
Beta-lactams	Cell wall biosynthesis	Piperacillin	R
Beta-lactams	Cell wall biosynthesis	Piperacillin/ Tazobactam	R
Fosfomycin	Cell wall biosynthesis	Fosfomycin	R
Glycopeptides	Cell wall biosynthesis	Teicoplanin	R
Glycopeptides	Cell wall biosynthesis	Vancomycin	R
Lincosamide	Protein synthesis	Clindamycin	63
Macrolides	Protein synthesis	Erythromycin	40
Nitrofuran	DNA replication/ Protein synthesis	Nitrofurantoin	30
Polymyxin	Structure of cell membrane	Colistin Sulphate	R
Polymyxin	Structure of cell membrane	Polymyxin B	33
Quinolones	DNA replication	Ciprofloxacin	68
Quinolones	DNA replication	Nalidixic	R
Tetracyclines	Protein synthesis	Doxycycline	12
Tetracyclines	Protein synthesis	Tetracycline	7

R resistant (no inhibition zone); Inhibition zone diameters, when present, are expressed in mm

## Conclusion

The polyphasic analysis including morphological, physiological and genomic features supports the results of the phylogenetic inference, which together delineate strain TO1_6^T^ from the known species of the genus *Stieleria*. Hence, we conclude that strain TO1_6^T^ represents the type strain of a new species of the genus, for which we propose the name *Stieleria tagensis* sp. nov.

### Description of *Stieleria tagensis* sp. nov.

#### *Stieleria tagensis* sp. nov. (ta.gen’sis. L. fem. adj. tagensis, pertaining to the Tagus River)

Cells are round to pear-shaped, with 1.45 ± 0.1 µm long and 1.02 ± 0.2 µm wide. Can occur as single cells or as rosettes and divide by budding. In solid medium, forms small, circular and white/beige colonies in the initial phase of incubation, that change to light pink-pigmented colonies. The temperature growth range is 15–30 °C, with optimal growth between 20 and 25 °C. Able to grow with 0–11% (w/v) NaCl, with optimal growth at 1%. Growth occurs from pH 5.0 to 9.5, with optimal growth at pH 7.0–7.5. Can grow without vitamins. Uses as nitrogen sources l-arginine, l-tyrosine, l-threonine, l-glutamine, l-isoleucine, l-aspartic acid, peptone, yeast extract, NAG and nitrate. Optimal growth was obtained with yeast extract. Can use as carbon source d-arabinose, cellobiose, glycerol, *myo*-inositol, lactose, maltose, d-manitol, d-sorbitol, saccharose, trehalose, d-xylose, d-glucose, raffinose and dextran. Optimal growth was obtained with dextran. Maximal doubling time is 17.2 h in M13 + NAG medium. Major fatty acids are C_16:0_ and C_18:1_ω9c Can grow under microaerophilic conditions, but not under anaerobic conditions. The genome size is 7.77 Mb with a DNA G + C content of 56.3%. The type strain is TO1_6^T^ (= CECT 30432^T^, = LMG 32465^T^), which was isolated from a river water sample from Tagus river in Portugal.

### Supplementary Information

Below is the link to the electronic supplementary material.

Supplementary file1 (PDF 420 kb)

Supplementary file2 (PDF 116 kb)

## Data Availability

The 16S rRNA gene sequence is deposited at NCBI’s GenBank database under accession number OK103954. The whole shotgun genome sequence is also deposited at NCBI’s GenBank under the accession number JAMYFE000000000.
