# Supplementary material for: Stieleria tagensis sp. nov., a novel member of the phylum Planctomycetota isolated from Tagus River in Portugal
Source: Antonie Van Leeuwenhoek. 2023 Sep 22;116(11):1209–25. doi: 10.1007/s10482-023-01877-2 (PMC10541342; doi:10.1007/s10482-023-01877-2)
Supplement: Supplementary file 1 — Supplementary file1 (PDF 420 kb) [file 10482_2023_1877_MOESM1_ESM.pdf]

Supplementary Table 2 - Results are the percentage of the total fatty acids. Values for fatty acids present in both strains at less than 0.5% are not shown; ECL= equivalent chain length. A summed feature represents groups of two or three fatty acids that could not be separated by GC with the MIDI System; --- = not detected

ECL*	Fatty acid	TO1_6^T Percentage	ICT_E10.1^T Percentage
14.002	$C_{14:0}$	0.87	0.29
15.206	$C_{14:0}$-2OH	0.56	---
15.818	Summed feature 3	9.03	6.62
16.002	$C_{16:0}$	31.33	31.75
16.794	$C_{17:1}$ω8c	2.9	0.66
17.001	$C_{17:0}$	1.12	0.32
17.576	$C_{18:3}$ω6c (6,9,12)	0.21	0.53
17.771	$C_{18:1}$ω9c	43.68	46.94
17.822	$C_{18:1}$ω7c	0.68	0.77
18.000	$C_{18:0}$	3.46	7.51
18.868	Summed feature 7	1.85	0.64
19.551	$C_{18:0}$-3OH	1.63	2.51
19.997	$C_{20:0}$	0.57	0.26

* ECL= equivalent chain length
Summed feature 3: $C_{16:1}$ω7c/iso $C_{15:0}$-2OH
Summed feature 7: cyclo-$C_{19:0}$ω10c/ 19ω6
