# Supplementary material for: Stieleria tagensis sp. nov., a novel member of the phylum Planctomycetota isolated from Tagus River in Portugal
Source: Antonie Van Leeuwenhoek. 2023 Sep 22;116(11):1209–25. doi: 10.1007/s10482-023-01877-2 (PMC10541342; doi:10.1007/s10482-023-01877-2)
Supplement: Supplementary file 2 — Supplementary file2 (PDF 116 kb) [file 10482_2023_1877_MOESM2_ESM.pdf]

Supplementary Table 1 - antiSMASH results the analysis of the genome of TO1_6^{T}.

Identified secondary metabolite regions using strictness 'relaxed'							MIBiG comparison (Analysis type: Protocluster to Region)				
Region	Type	From	To	Most similar known cluster		Similarity	Reference	Similarity score	Type	Compound(s)	Organism
Region 7.1	T3PKS	5,291	35,365				BGC0001962.1	0.36	Polyketide	hierridin B, hierridin C	Cyanobium sp. LEGE 06113
Region 8.1	NRPS-like	12,601	56,062	nematophin	NRP	12%	BGC0001831.1	0.18	Polyketide	alkylpyrone-407, alkylpyrone-393	Myxococcus xanthus DK 1622
Region 18.1	terpene	3,218	24,507				BGC0000647.1	0.32	Terpene	carotenoid	Rhodobacter sphaeroides
Region 34.1	terpene	847	16,581				BGC0000272.1	0.18	Polyketide	spore pigment	Streptomyces collinus
Region 136.1	NRPS,T1PKS	18,927	69,499				BGC0001187.1	0.69	NRP, Polyketide	xenolozoyenone	Glarea lozoyensis
Region 141.1	acyl_amino_acids	174	97,962				BGC0001591.1	0.33	Other	fatty acid enol ester	uncultured bacterium CSLC2
Region 182.1	T1PKS	34,555	83,239				BGC0001197.1	0.24	Polyketide	frankiamicin	Frankia sp. EAN1pec
